# Increased novelty-induced locomotion, sensitivity to amphetamine, and extracellular dopamine in striatum of *Zdhhc*15-deficient mice

**DOI:** 10.1038/s41398-020-01194-6

**Published:** 2021-01-18

**Authors:** Rebeca Mejias, Juan J. Rodriguez-Gotor, Minae Niwa, Irina N. Krasnova, Abby Adamczyk, Mei Han, Gareth M. Thomas, Zheng-Xiong Xi, Richard L. Huganir, Mikhail V. Pletnikov, Akira Sawa, Jean-Lud Cadet, Tao Wang

**Affiliations:** 1 McKusick-Nathans Institute of Genetic Medicine and Department of Pediatrics, Johns Hopkins University School of Medicine, Baltimore, MD 21205 USA; 2 Department of Physiology, University of Seville, 41012 Seville, Spain; 3 Department of Psychiatry and Behavioral Sciences, Johns Hopkins University Bloomberg School of Public Health, Baltimore, MD 21205 USA; 4 Department of Psychiatry and Behavioral Neurobiology, University of Alabama, Birmingham, AL 35233 USA; 5 Molecular Neuropsychiatry Research Branch, Intramural Research Program, NIDA/NIH/DHHS, Baltimore, MD 21224 USA; 6 Division for Research Capacity Building, NIGMS/NIH/DHHS, Bethesda, MD 20892 USA; 7 Department of Anatomy and Cell Biology, Temple University, Philadelphia, PA 19140 USA; 8 Shriners Hospitals Pediatric Research Center, Temple University, Philadelphia, PA 19140 USA; 9 National Institute on Drug Abuse, Intramural Research Program, Baltimore, MD 21224 USA; 10 The Solomon H Snyder Department of Neuroscience, Johns Hopkins University School of Medicine, Baltimore, MD 21205 USA; 11 Department of Psychological and Brain Sciences, Johns Hopkins University, Zanvyl Krieger School of Arts & Sciences, Baltimore, MD 21218 USA; 12 Departments of Psychiatry, Neuroscience, and Biomedical Engineering, Johns Hopkins University School of Medicine, Baltimore, MD 21205 USA; 13 Department of Mental Health, Johns Hopkins University Bloomberg School of Public Health, Baltimore, MD 21205 USA

**Keywords:** Molecular neuroscience, ADHD

## Abstract

Novelty-seeking behaviors and impulsivity are personality traits associated with several psychiatric illnesses including attention deficits hyperactivity disorders. The underlying neural mechanisms remain poorly understood. We produced and characterized a line of knockout mice for *zdhhc15*, which encodes a neural palmitoyltransferase. Genetic defects of *zdhhc15* were implicated in intellectual disability and behavioral anomalies in humans. *Zdhhc15*-KO mice showed normal spatial learning and working memory but exhibited a significant increase in novelty-induced locomotion in open field. Striatal dopamine content was reduced but extracellular dopamine levels were increased during the habituation phase to a novel environment. Administration of amphetamine and methylphenidate resulted in a significant increase in locomotion and extracellular dopamine levels in the ventral striatum of mutant mice compared to controls. Number and projections of dopaminergic neurons in the nigrostriatal and mesolimbic pathways were normal. No significant change in the basal palmitoylation of known ZDHHC15 substrates including DAT was detected in striatum of *zdhhc15* KO mice using an acyl-biotin exchange assay. These results support that a transient, reversible, and novelty-induced elevation of extracellular dopamine in ventral striatum contributes to novelty-seeking behaviors in rodents and implicate ZDHHC15-mediated palmitoylation as a novel regulatory mechanism of dopamine in the striatum.

## Introduction

Attention deficit hyperactivity disorder (ADHD) is a heterogeneous neurodevelopmental disorder affecting 7.2% of children^1^ and 3.4% of adult populations^2^. It is characterized clinically by two core symptoms, inattention (IA) and hyperactivity–impulsivity (HI), with a high heritability at 70–80%^3–6^. Novelty seeking is a core dimension of temperament such as exploratory excitability and impulsivity^7^. Previous studies consistently showed that patients with ADHD score highly for novelty-seeking behaviors^8–15^. Accumulating evidence suggests that novelty seeking is genetically associated with and is predictive of high scores for IA and HI symptoms in ADHD^9,12,15^. Neurochemical studies of a high novelty responsive rat model identified a distinct DA metabolic profile and a higher basal and extracellular dopamine levels in the nucleus accumbens compared with low responsive ones^16,17^. Dysregulations of dopaminergic signaling in the substantia nigra/ventral tegmental area (SN/VTA) were implicated^18,19^ but the precise regulatory mechanisms are poorly understood.

Protein palmitoylation is a key post-translational modification characterized by a reversible addition of palmitate at specific cysteine residues. Palmitoylation can impact membrane association, trafficking and intracellular localization, protein–protein interactions, enzyme activity, and stability of substrate proteins^20,21^. Palmitoylation reaction is catalyzed by a family of palmitoyl-transferases (PATs, *n* = 23) sharing a conserved Asp-His-His-Cys (DHHC) motif in mammals^20,22^. Palmitoylation plays important roles in normal physiology and diseases including schizophrenia, anxiety disorders, intellectual disability, cancer, mitochondrial functions, eye and heart development, and endothelial inflammation^23–28^.

Approximately 41% of known synaptic proteins are predicated to be substrates of palmitoylation^29^, which highlights the importance of palmitoylation in synaptic development and functions^30–33^. In the dopaminergic signaling pathway, it has been shown that palmitoylation of dopamine transporter (DAT) is enhanced by co-transfection of *DAT* with individual expression constructs for *zdhhc2*, *zdhhc3*, *zdhhc8*, *zdhhc15*, or *zdhhc17* in an ABE assay^34^. Abolishing DAT palmitoylation by altering a key cysteine residue, Cys580, leads to reduced DA efflux and instability of DAT protein^34–36^. Additional proteins in dopamine signaling and/or metabolism including dopamine receptors 1–4 and G protein α subunits were also found palmitoylated in vitro but the responsible PATs are not determined^37–41^. Dysregulations of DA homeostasis are implicated in Parkinson disease, schizophrenia, bipolar disorder, major depression, ADHD, and drug addiction^42,43^.

*Zdhhc15*, located at Xq13.3 in humans, encodes a protein palmitoyltransferase that is highly expressed in brain tissues^44–46^. *Zdhhc15* knockdown was shown to reduce number and maturation state of dopaminergic neurons in zebrafish^44^ and to decrease dendritic outgrowth, arborization, and spine maturation in cultured rat hippocampal neurons^45^. In vitro palmitoylation assays identified multiple ZDHHC15 substrates including PSD95 (postsynaptic density protein 95), CSP (Cysteine String Protein), DAT, GAP-43 (Growth Associated Protein 43), SNAP25b (Synaptosome Associated Protein 25, variant b), Sortilin, and Stathmin-2^47^. An X-autosome translocation disrupting *zdhhc15* expression was found in a female with intellectual disability^48^. However, the roles of *zdhhc15* genetic defects in cognitive and psychiatric disorders in humans remain to be established^49,50^.

We produced and functionally characterized a line of *zdhhc15*-KO mice that exhibit a significant increase in novelty-induced locomotion and enhanced sensitivity to amphetamine. We showed that a transient, reversible, and novelty-induced elevation of extracellular dopamine in ventral striatum correlates with the novelty-seeking behaviors in mutant mice. Our data implicate ZDHHC15 palmitoylation as a novel regulatory mechanism for striatal dopamine and novelty-seeking behaviors in rodents. Our results provide valuable insights into pathological mechanisms involved in dopaminergic alterations in neuropsychiatric disorders such as ADHD.

## Materials and methods

### Generation and genotyping of *zdhhc*15 knockout mice

ES cell line, AH0619, containing a gene-trap in *zdhhc15* gene, was obtained from the Wellcome Trust Sanger Institute (Cambridge, Hinxton, UK). *Zdhhc15* knockout (*zdhhc15*-KO) chimeric mice were generated at the Embryonic Stem Cell Core Facility (Johns Hopkins University, Baltimore, USA) by standard microinjection of the AH0619 ES cells into blastocysts. Chimeric males were crossed with wild type (WT) C57BL/6J females (The Jackson Laboratory, Bar Harbor, Maine) to generate F1 offspring on a mixed 129P2/OlaHsd and C57BL/6J background. *Zdhhc15*-KO mice have been back-crossed to C57BL/6J background for >10 generations to achieve isogenic background.

Similar to that in humans, *zdhhc15* was mapped to the X chromosome in mice. Male hemizygous *zdhhc15*-KO and WT littermates were obtained for the experiments by breeding heterozygous females with C57BL/6J WT males. Confirmation of gene-trap insertion resulting in a loss of *zdhhc15* expression was performed by RT-PCR using one-step RT-PCR kit (Qiagen) using the following set of primers: M3: 5′-CAGCCAAACCAGAAGTTCCACTTG-3′ (forward), M4: 5′-CTGGGTAATTCCCCTCTCCAG (reverse), and B-geo-S1: 5′-AGTATC GGCCTCAGGAAGATCG-3′ (reverse). Further confirmation of location of the gene-trap (12,249 pb position in intronic sequence between exons 4 and 5) was determined by PCR amplification of genomic DNA from a *zdhhc15*-KO mouse, using the primers: IN-12013-F: 5′-TCTGAGCCAAATCCAAGC-3′ (forward) and ZDH-seq-AA1: 5′-CCTTGGGACCACCTCATCAGAAG-3′ (reverse), and subsequently standard Sanger sequencing. PCR primers used for genotyping consist of ZDH-En2-F: 5′-GTTGGTTGTGGATAAGTAGCTAGACT-3′ (forward) and B-geo-S1-R: 5′-AGTATC GGCCTCAGGAAGATCG-3′ (reverse) for amplifying the KO allele (∼400 bp), and IN-12013-F: 5′-TCTGAGCCAAATCCAAGC-3′ (forward) and IN-12483-R: 5′-TAGCAACAGAGCAGAGAACG-3′ (reverse) for the WT allele (∼500 bp). All experimental procedures with mice were approved by the Animal Care and Use Committee of the Johns Hopkins University School of Medicine and the Institutional Committee of the University of Seville for Animal Care and Use (CEEA-US2016-15/4), and were done in compliance with federal and state laws and policies (Maryland, USA) and the European Community Council directives 86/609/EEC, and 2010/63/EU for the Care and Use of Laboratory Animals. Rodent housing room was maintained at 23 °C on a 12-h light/dark cycle (9:00 and 21:00). All experimental animals were provided with free access to standard mouse chow and water.

### Immunohistochemistry and densitometric assay of striatal TH immunoreactivity and stereological cell counting

Age-matched WT and *zdhhc15*-KO males (1-year old) were perfused with PBS and 4% paraformaldehyde/PBS, pH 7.4. Brains were removed and post-fixed 4 h in the same fixative. After cryoprotection in 30% sucrose/PBS, pH 7.4, brains were frozen and 30-μm serial coronal sections were cut using a sliding microtome. Free-floating sections were blocked with 4% goat serum/PBS plus 0.2% Triton X-100, incubated with an antibody against tyrosine hydroxylase (anti-TH rabbit polyclonal, Novus Biologicals), and then treated with biotin-conjugated anti-rabbit antibody (goat polyclonal, Jackson ImmunoResearch), ABC reagent (Vector Laboratories), and SigmaFast DAB Peroxidase Substrate (Sigma). The sections were then counterstained with Nissl (0.09% thionin) followed by destaining with 1% formalin acetic acid and mounted with Permount^TM^ Media (Fisher Scientific). Densitometric measurements of TH signal were carried out for each animal from three pictures from TH-immunostained striatal sections using the ImageJ software. Density of the cortex was used to subtract the general background. Stereological cell counts of TH-positive neurons from the substantia nigra pars compacta (SNpc) and the ventral tegmental area (VTA) were estimated by systematic random sampling using the optical fractionator method^51^ with a newCAST^TM^ software (Visiopharm) installed into a BX61- Olympus microscope. The mean cell volume of TH+ neurons was quantified using the rotator method^52^ with the same software.

### Mouse behavioral testing

Age-matched WT and *zdhhc15*-KO males (2–4 months) were tested at the Johns Hopkins University Rodent Behavioral Core following the standard protocols (http://www.brainscienceinstitute.org/index.php/cores). Mouse behavioral testing was carried out in ambient light at the same time period if more than 1 day of testing are needed. Behavioral data were analyzed by two independent investigators who are blind to the genotypes of the experimental animals.

#### Open-field test

Each mouse was placed for 30, 60, or 90 min in the open-field chamber, a 45 × 45-cm clear plastic chamber equipped with photo-beams (*n* = 16 at equal spacing of 2.5 cm). Their movements were tracked using the SDI Photobeam Activity System (San Diego Instruments). Overall activity, fine movements, rearing movements, and activity in the center and periphery of the chamber were determined. The peripheral area (425 cm^2^) was defined by the two side-photo beams, #1–2 and #15–16, while the central area (1600 cm^2^) was defined by photo beams #3–14 at each direction.

#### Sociability and preference for social novelty

Social tests were carried out in a 45 cm (*L*) × 45 cm (*W*) × 37.5 cm (*H*) clear plastic chamber with two small mesh cages (10 cm in diameter, 15 cm high) placed at opposite corners, and divided equally into four quadrants. The test mouse was allowed to explore the chamber and mesh cages freely for 5 min just before the start of the trials. For trial 1 (sociability), a reference stranger male mouse (C57BL/6) was placed inside one of the mesh cages and the test mouse was allowed to explore the chamber freely for 10 min. For trial 2 (social novelty), a second C57BL/6 stranger mouse was placed in the other mesh cage and the test mouse was allowed to explore freely for another 10 min. The time that the experimental mouse spent in each of the four quadrants was measured for both 10 min sessions.

#### Elevated-plus maze

The elevated-plus maze (San Diego Instruments) has two closed arms and two open arms connected by a middle platform. The closed arms measure 48 cm in length × 10 cm in width × 38 cm in height and the open arms measure 48 cm in length × 10 cm in width. The test mouse was placed on the middle platform and was allowed to explore the maze freely for 5 min. The total time spent in the closed and open arms was recorded.

#### Y-maze of spontaneous alternations and blocked arms

The Y-maze consists of three identical arms of 46 cm (*L*) × 6.25 cm (*W*) × 2.5 cm (*H*), radiating from a central platform at 120° angles. The test was carried out in three trials. In the first trial, the test mouse was placed at the end of one arm (randomly chosen) and remained in the maze for 5 min. The total number of spontaneous alternations divided by the number of total possible alternations was recorded and analyzed. Seven days after the first trial, second and third trials were run. During the second trial, one of the arms, chosen randomly, was blocked. The test mouse was allowed to explore the two remaining unblocked arms for 5 min followed by a rest of 10 min. In the third trial, the test mouse was returned to the maze with all three arms open and allowed to explore for another 5 min. The third trial was analyzed for time spent in the arm that was blocked at second trial, for the first 2 min and full 5 min of trial three.

#### Morris water maze

A standard water maze (120 cm in diameter), containing opaque water (25 °C), was set up with a rescuing platform (10 cm × 10 cm) just below the water surface and marked with a cue (flag) and four large spatial cues outside the maze. On the first day of test, mice were subjected to four swimming trials of 1 min in the maze to locate the platform. In each of those four trials, the platform was placed at a different quadrant of the maze. On test days 3–7, the platform cue was removed and the mice were subjected to three trials of 1 min to locate the hidden rescuing platform placed at a fixed quadrant of the maze. During the final trial on day 11, mice were subjected to 3 min of free swimming in the water maze without platform. The time spent in the quadrant where the platform was located on days 8–10 was determined and analyzed. For reverse water maze, the animals were trained and tested again in the same way than for the standard water maze but the scape platform was fixed to the opposite quadrant to its previous position.

#### Prepulse inhibition

SR-LAB startle response system (San Diego Instruments) was used to determine the prepulse inhibition response. The test mouse was placed inside of a startle chamber and a 70-dB background noise level was presented for the acclimation period (5 min) and continued throughout the test trial. Each mouse was subjected to six sets of seven trial types distributed pseudo randomly: pulse-alone trials, prepulse-pulse trials, and no-stimulus trials. The pulse alone used was 120 dB and the prepulses were 74, 78, 82, 86, and 90 dB. The interval between stimuli was 10 s and the intertrial intervals (assigned pseudo randomly) were 40–70 s. The maximum startle response was recorded and normalized by the body weight of the test mouse. The percent of prepulse inhibition was calculated by dividing the startle response from each prepulse trial by the mean startle response from pulse-only trials.

#### Rotarod test

A Rotamex-5 with mouse spindle (Columbus Instruments) was used to measure motor coordination. The test mouse was placed on the rotating rod accelerating from 5 to 30 rpm during a 5-min session, and the time on the rod before falling was recorded. Each mouse was tested under the same parameters three times each day for 3 days. Data from all nine sessions were obtained and analyzed for each mouse.

#### Fear-conditioning test

The testing chamber consisted of a conditioning arena located inside of a sound attenuating shell. Each chamber has an electrifiable floor grid, speakers mounted on the wall, and a red ambient light. The neutral conditioning stimulus (CS, cue conditioning) consisted of an 80 dB 2 kHz tone lasting 20 s. During every session, a period of 30 s of habituation preceded the presentation of the cue (20 s). The aversive unconditioned stimulus (US) consisted of a 0.5 mA current lasting 2 s and was delivered during the last 2 s of the tone, so CS and US were co-terminating (paired conditioning). Each trial consisted of 6 sessions (total time 300 s). Each mouse was tested under the same parameters for three trials, trial 1; trial 2, 24 h after trial 1; and trial 3, 1 week after trial 2. The total amount of freezing time on a trial was calculated for each test mouse.

#### Novel object recognition test

The novel object recognition test was carried out in a black plexiglass open-field arena (25 cm × 25 cm). Each individual mouse was allowed to explore the arena for 10 min without objects each day for a total of 3 consecutive days of acclimation (habituation phase). On day 4, two identical novel objects were secured on the center of the arena’s floor and each individual mouse was allowed to explore the arena for 5 min (training phase). Time spent exploring each object was recorded. On the same day, 1 h after the training, one of the familiar objects used during the training phase was replaced by a novel object similar but not identical in size and shape. Each animal was allowed to explore for 5 min and the time spent exploring each object was recorded (retention phase).

### Acyl-biotinyl exchange (ABE) assay

Palmitoylation levels of proteins were detected by using an acyl-biotinyl exchange (ABE) assay. ABE analysis of proteins was performed as published previously^53^ with minor modifications. Dissected parietal cortex or striatum was homogenized using a polytron in lysis buffer (50 mM HEPES, 2% SDS, 1 mM EDTA) supplemented with 1% Triton, 20 mM MMTS (methyl methanethiosulfonate, Thermo Scientific) and protease inhibitors (Complete Mini EDTA-free, Roche Diagnostics). Cell debris was pelleted by centrifugation at 2000 × *g* and supernatant was incubated 20 min at 37 °C to block free thiols. Proteins were precipitated using acetone followed by thioester cleavage using 0.7 M of hydroxylamine (SIGMA) and labeled with 1 mM biotin-HPDP (Soltec Ventures) for 1 h at room temperature. Proteins were precipitated again with acetone. Biotinylated proteins were pulled down by binding with neutravidin-agarose (Thermo Scientific) and analyzed by western blot.

### Western blot analysis

Flash-frozen tissue was homogenized with a polytron in lysis buffer (1% Nonidet P-40, 10% glycerol, 137 mM NaCl, 20 mM TrisHCl, pH 7.4) supplemented with protease inhibitors (Roche). After incubation on ice for 30 min, the homogenized samples were centrifuged at 14,000 rpm for 10 min at 4 °C and supernatants were used for western blotting. Proteins were separated by electrophoresis in Bis-Tris gels and transferred onto PDVF membranes using standard procedures. Staining of proteins contained in membranes was carried out with Ponceau S Red (Sigma-Aldrich). Membranes were incubated with the following primary antibodies: acetylated alpha-tubulin (mouse, Santa Cruz Biotechnology); COMT (rabbit, Abcam); CSP (rabbit, Enzo); DA beta hydroxylase (rabbit, Abcam); DAT (rat, Millipore); DRD1 (rabbit, Millipore); DRD2 (rabbit, Millipore); GAP43 (rabbit, Millipore); MAO-B (rabbit, Abcam); PSD95 (mouse, NeuroMab); Snap25 (rabbit, Abcam); Sortilin (rabbit, Abcam); Stathmin-2 (mouse, NeuroMab); TH (rabbit, Novus Biologicals); ZDHHC15 (affinity-purified rabbit polyclonal antibody against the mouse sequence peptide: YTDKERYKNEERPEC, GenScript Corporation); followed by horseradish peroxidase-conjugated secondary antibodies. Bands were visualized with western blotting detection reagent (GE Healthcare) by exposition to X-ray films or in a chemiluminescence image system (Fusion Solo S, Vilber), and signal was quantified using ImageJ (NIH).

### Quantitative monoamine analysis

Monoamine concentrations were analyzed in homogenates from discrete brain regions. Tissues were sonicated in chilled 0.1 M perchloric acid and centrifuged at 15,000 × *g* for 20 min at 4 °C. NE, DA, DOPAC, HVA, 5-HT, and 5-HIAA levels were analyzed in 10 μl of the supernatants obtained using HPLC with electrochemical detector as previously described^54^. Data were normalized by tissue weight.

### In vivo microdialysis assay

Microdialysis was carried out as previously described^55^ with minor modifications. Briefly, a guide cannula was implanted into the nucleus accumbens (AP: +1.7 mm, ML: −0.8 mm from Bregma, DV: −4.0 mm from the dura) according to the atlas of Franklin and Paxinos. Artificial CSF (147 mM NaCl, 2.8 mM KCl, 1.2 mM CaCl_2_, 1.2 mM MgCl_2_) was perfused at a flow rate of 1.0 μl/min. The dialysates were collected every 10 min and analyzed by an HPLC system. Six samples were taken to establish basal levels of extracellular dopamine (WT, 0.67 ± 0.05 nM; ZDHHC15-KO, 0.62 ± 0.06 nM). The extracellular levels of dopamine during habituation phase (30 min), baseline phase (60 min), and amphetamine (2.0 mg/kg, i.p.) phase (120 min) were measured in the nucleus accumbens.

### Drug treatment

Mice were given i.p. injection of the indicated drugs and then placed in the open-field chamber immediately. Mice received a single dose injection (i.p.) of methylphenidate (Ritalin; 1.5, 2.5, or 4 mg/kg) or d-amphetamine (0.5, 1.0, or 2.0 mg/kg) dissolved in saline (0.9% NaCl). Saline without the drug was administered as vehicle control. A washout period of at least 7 days was left between treatments.

### Statistical analysis

Statistics analyses were performed using unpaired two-tailed *t*-test for comparison of the means of two groups, and factorial repeated-measures ANOVA followed by Bonferroni *post hoc* test for multiple comparisons between groups when subjects were measured repeatedly in time. We used factorial ANOVA followed by Bonferroni *post hoc* test to compare more than one independent variable. Analyses were performed with SPSS Statistics Software. Specific test used to analyze data included in figures is indicated in the figure legends. Significance is reported at *p* ≤ 0.05, and data at graphs and tables represent the mean values ± s.e.m.

## Results

### Generation and genetic characterization of *zdhhc*15 KO mice

**Fig. 1:**
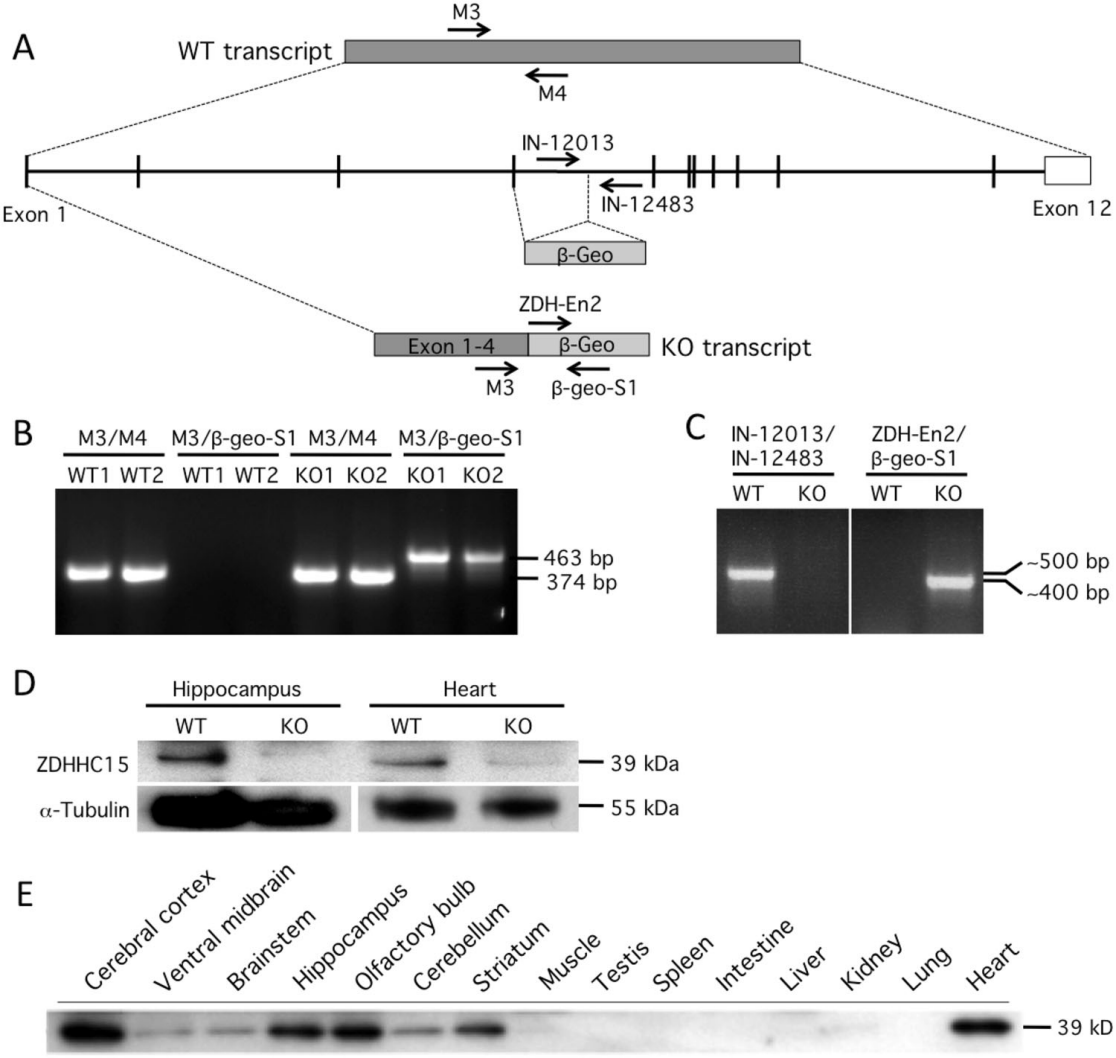
Generation and characterization of *zdhhc15*-KO mice. **A** *Zdhhc15*-KO mice were generated by standard microinjection using an ES cell line containing a gene-trap in the *zdhhc15* gene from the Wellcome Trust Sanger Institute, Cambridge, UK. **B** Confirmation of insertion of the gene-trap into the *zdhhc15* transcript in the ES cells was performed by standard RT-PCR using the primers, M3, M4, and β-geo-S1. M3 and M4 amplified the shared region of *zdhhc15* transcript; M3 and β-geo-S1 detected the gene-trapped transcript. **C** Genotyping of *zdhhc15*-KO mice was carried out using the primers IN-12013-F (forward) and IN-12483-R (reverse) for the WT allele (~500 bp), and ZDH-En2-F (forward) and B-geo-S1-R (reverse) for amplifying the KO allele (~400 bp). **D** Immunoblot of ZDHHC15 using a custom rabbit antibody against mouse sequence peptide: YTDKERYKNEERPEC (GenScript Corporation) showing almost non-detectable levels of protein in hippocampus and heart of the *zdhhc15*-KO mice. **E** Tissue expression profile of ZDHHC15 in a WT mouse. Immunoblot using the same custom-rabbit antibody against ZDHHC15 (GenScript Coorporation) identified a 39 kD band corresponding to ZDHHC15 protein that was highly expressed in brain regions and heart of WT mice.

*Zdhhc15* knockout (KO) mice were generated by microinjection into blastocysts of an ES cell line containing a gene-trap into the *zdhhc15* gene (Fig. 1A). Confirmation of the insertion of the gene-trap into the *zdhhc15* transcript was performed by standard RT-PCR using primers M3, M4, and B-geo-S1 (Fig. 1B). Further confirmation of location of the gene-trap between exons 4 and 5 was determined by PCR amplification and sequencing of genomic DNA from male *zdhhc15*-KO mice (data not shown). Offspring were genotyped by PCR using primers IN-12013 and IN-12483 to amplify the WT allele (∼500 bp) and ZDH-En2 and B-geo-S1 for the KO allele (∼400 bp) (Fig. 1C). Near complete lack of ZDHHC15 protein in hippocampus and heart (tissues with high levels of *Zdhhc15* expression, see Fig. 1E) of KO mice was confirmed by western blot analyses (Fig. 1D) using a custom rabbit polyclonal antibody (affinity-purified antibody produced by GenScript Corporation). The pattern of expression for ZDHHC15 protein in the western blot analyses (Fig. 1E) was in accordance to previous studies of the mRNA levels of this gene in different tissues^46^.

### *Zdhhc15*-KO mice exhibit normal learning and memory functions

*Zdhhc15* has previously been implicated in intellectual disability in humans^48^. Using standard rodent behavioral tests, we evaluated learning and memory functions in male *zdhhc*15-KO mice. Compared to male WT littermates, mutant mice show normal working memory function in Y-maze of spontaneous alternation and closed arm test, normal spatial learning in Morris water maze, normal fear memory in fear-conditioning test, and normal recognition memory in novel object recognition test (Supplementary Table 1).

### *Zdhhc15*-KO mice show normal anxiety levels and social interactions

*Zdhhc*15-KO mice were evaluated for anxiety-related behaviors and social interactions. Compared to WT male littermates, mutant mice showed normal anxiety levels in elevated-plus maze and normal sociability and preference in social novelty using standard social behavioral tests (Supplementary Table 1).

### *Zdhhc15*-KO mice show a novelty-induced increase in ambulatory activities

**Fig. 2:**
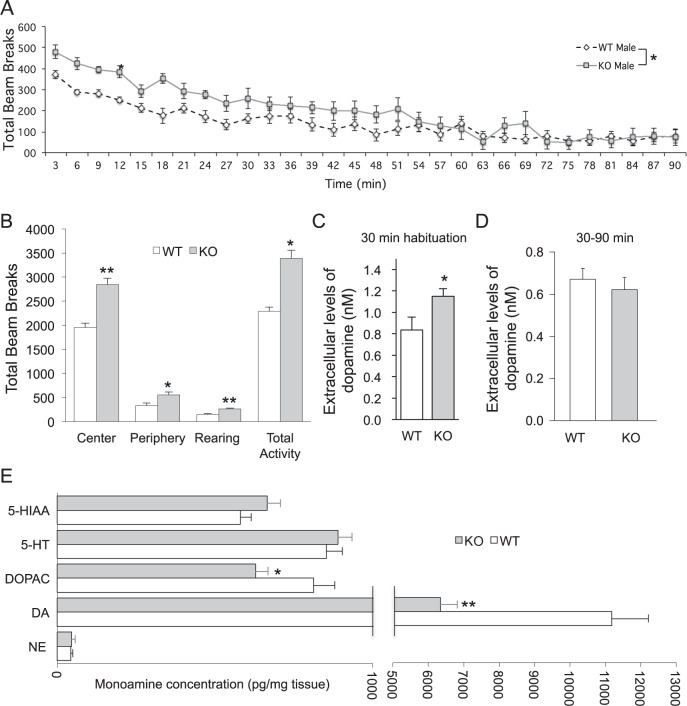
Novelty-associated increase in locomotion and tissue and extracellular dopamine levels in ventral striatum of *zdhhc15*-KO mice. **A** Individual male *zdhhc15*-KO mice and matched WT littermates were allowed to explore freely from interference for 90 min in a photo-beam equipped clear plastic chamber. The patterns of total ambulatory activity were automatically recorded and analyzed. Adult male *zdhhc15*-KO mice show increased motor activity when compared to WT littermates (*n* = 9–10 animals per group; factorial repeated measures ANOVA; *p* = 0.023). The increased locomotion in *zdhhc15*-KO was most significant during the first 30 min in the chamber, gradually reduced to levels of WT mice during 30–60 min, and remained at levels of WT mice during 60–90 min). **B** Significant increase in motor activities in male *zdhhc15*-KO mice was observed during the first 30 min (habituation phase) of the test (*n* = 9–10 animals per group; student *t*-test; center, *p* = 0.00002; periphery, *p* = 0.01536; rearing, *p* = 0.000027). **C** Adult male *zdhhc15*-KO mice show increased extracellular dopamine as measured by in vivo microdialysis in the ventral striatum during the habituation phase (30 min) to the novel environment, which corresponds to the increase in locomotion observed in the first 30 min at the open-field test (*n* = 6 mice per group, student *t*-test, *p* = 0.049). **D** After the habituation phase, the extracellular levels of dopamine in ventral striatum were not significantly different between WT and *zdhhc15*-KO mice (*n* = 6 mice per group). **E** Striatal content of monoamines in male *zdhhc15*-KO and WT control mice as measured by HPLC. NE, norepinephrine; DA, dopamine; DOPAC, 3,4-Dihydroxyphenylacetic acid; HVA, homovanillic acid; 5-HT, serotonin; 5-HIAA, 5-hydroxyindoleacetic acid (*n* = 10 mice per group, student *t*-test, *p < 0.05, **p < 0.01).

In standard open-field test, *zdhhc*15-KO mice show a significant increase in ambulatory activities that occur in both central and peripheral regions of the test chamber (Fig. 2A, B). This increase in ambulatory activities appears to be novelty-induced. It peaks during the first 30 min, sustains over 60 min, and returns to baseline levels during 60–90 min in the test chamber compared to WT controls (Fig. 2A, B). Furthermore, *zdhhc15*-KO mice do not exhibit hyperactivity in their home cages (data not shown). These data suggest that the hyperactivity phenotypes in *zdhhc15*-KO mice correlate with their exposure to a novel environment and, once the test animals are familiarized with their surroundings, their activity levels return to baseline similar to WT controls. We detected no differences in motor coordination in the rotarod test, or in sensorimotor gating and integration in Prepulse Inhibition (PPI) test between *zdhhc*15-KO mice and matched WT littermates (Supplementary Table 1).

### Increased extracellular dopamine levels in ventral striatum during hyperactivity

Because hyperactivity has been linked before to decreased total tissue content of DA but increased extracellular DA levels in rodents^56,57^, we determined extracellular DA levels in ventral striatum of *zdhhc15*-KO mice and matched WT littermates by microdialysis and HPLC during different phases in an open field (Fig. 2C, D). During the first 30 min or habituation phase, we observed a significant increase in extracellular DA levels in ventral striatum when the mutant mice exhibited a significant increase in ambulatory activities (Fig. 2C). In contrast, during the period of 30–90 min in open field when the ambulatory activities of the mutant mice returned to baseline, we found comparable extracellular DA levels in ventral striatum between WT and *zdhhc15*-KO mice (Fig. 2D). Therefore, this data shows that the hyperactivity observed in the *zdhhc15*-KO mice during the habituation phase of the open-field test is associated with an increase in extracellular DA in the ventral striatum, and once this phase ends, the extracellular dopamine levels go back to baseline as the WT mice.

### Reduced tissue dopamine and its metabolites in striatum of *zdhhc15*-KO mice

To further study the motor phenotype found in *zdhhc15*-KO mice, we quantified the monoamine content in different brain regions of male KO and WT mice by electrochemical detection using a HPLC. We measured norepinephrine, dopamine, and serotonin in prefrontal cortex, olfactory bulb, striatum, and ventral mesencephalon in *zdhhc15*-KO mice and matched WT littermates. Intriguingly, we observed a significant reduction of tissue dopamine levels and its metabolite, DOPAC, specifically in striatum of *zdhhc*15-KO mice as compared to WT littermates (Fig. 2E). No difference was detected in tissue monoamine levels in other brain regions (Supplementary Table 2) except metabolite of serotonin, 5-HIAA, was noted to be slightly increased only in olfactory bulb without a significant change in total serotonin (5-HT) levels (Supplementary Table 2).

### Normal number and morphology of DA neurons in *zdhhc15*-KO mice

**Fig. 3:**
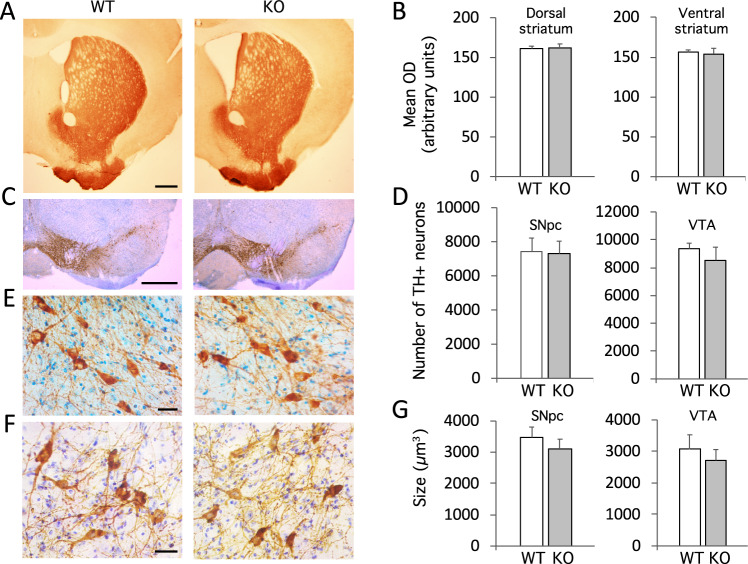
Cell body size and number of dopaminergic neurons in the nigrostriatal and mesolimbic systems from male *zdhhc15*-KO mice. **A** Representative images of striatal sections immunostained using an anti-TH antibody from WT and *zdhhc15*-KO mice. Scale bar: 500 μm. **B** There were no significant differences between WT and KO mice in the density of projections of dopaminergic neurons to the dorsal or ventral striatum. Measurements of the mean density were performed using ImageJ in pictures at three different Bregma levels in the striatum. **C** Representative images of coronal sections of mesencephalon from WT and *zdhhc15*-KO mice after TH immunostaining. Scale bar: 50 μm. **D** Quantification of the number of TH+ neurons in the SNpc and VTA from *zdhhc15*-KO mice and controls. There were no significant differences between WT and KO mice in the number of dopaminergic neurons in the SNpc or the VTA quantified by stereological measurements. **E**, **F** Representative images showing dopaminergic neurons stained against TH in the SNpc (**E**) and VTA (**F**) of WT and KO mice. Scale bar: 10 μm. **G** There were no significant differences between WT and KO mice in the size of the cell body of dopaminergic neurons in the SNpc. Quantitation was performed by stereology using the rotator method (*n* = 6 mice per genotype, student *t*-test, *p* > 0.05).

To assess whether the marked decrease of tissue DA levels in striatum of *zdhhc15*-KO mice is due to a loss of dopaminergic neurons of the nigrostriatal and/or mesolimbic pathways, we carried out stereological counting of tyrosine hydroxylase (TH)-positive neurons in the substantia nigra pars compacta (SNpc) and ventral tegmental area (VTA), and conducted a densitometric study of their projections to dorsal and ventral striatum. The number of dopaminergic neurons and the TH-density signal in striatum were not significantly different between *zdhhc15*-KO mice and their WT littermates at 1 year of age (Fig. 3A–D). We also quantified the mean cell volume of TH+ cells in the SNpc and VTA of KO and WT animals and found no differences between both genotypes (Fig. 3E–G). Therefore, we found no evidence of degeneration of DA neurons in the nigrostriatal and/or mesolimbic pathways that could explain the reduced tissue DA levels in striatum of *zdhhc15*-KO mice, suggesting that altered DA release and/or reuptake and metabolism are likely responsible.

### *Zdhhc15*-KO mice show enhanced sensitivity to psychostimulants

**Fig. 4:**
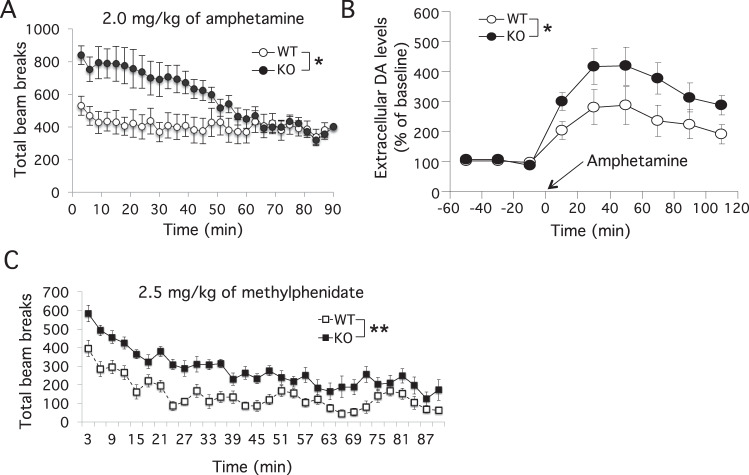
Enhanced locomotion in response to amphetamine and methylphenidate treatment for adult male *zdhhc15*-KO mice. Mice were first given i.p. injection of the indicated drugs and then placed in open-field chamber immediately for measurement of locomotion. **A** Amphetamine treatment of *zdhhc15*-KO mice resulted in higher levels of locomotion compared to WT controls in open field and this effect was most prominent during the first 60 min (2.0 mg/kg, i.p. injection at time = 0 min; *n* = 9–10 animals per group, factorial repeated measures ANOVA, *p* = 0.03). **B** Z*dhhc15*-KO mice showed an increase in extracellular dopamine level in ventral striatum in response to amphetamine. The extracellular levels of dopamine during baseline phase (60 min), and amphetamine (2.0 mg/kg, i.p.) phase (120 min) were measured in the nucleus accumbens by microdialysis (*n* = 6 mice per group, factorial repeated measures, ANOVA, *p* = 0.047). **C** Methylphenidate treatment resulted in an increase in locomotion in *zdhhc15*-KO mice compared to WT controls (2.5 mg/kg, i.p. injection at time = 0 min; *n* = 9–10 animals per group, factorial repeated measures ANOVA, *p* = 0.0001).

Psychostimulants such as methylphenidate and amphetamine are known to modulate DA release and/or reuptake at synapses and are used to treat hyperactivity phenotype in animal models and patients with attention deficits hyperactivity disorders^58^. We tested these drugs on activity and extracellular DA levels in *zdhhc15*-KO mice. Adult male *zdhhc15*-KO mice and matched WT littermates were given a single i.p. injection of d-amphetamine (0.5, 1.0, or 2.0 mg/kg) or methylphenidate (Ritalin; 1.5, 2.5, or 4 mg/kg) and were tested individually in an open-field test. We observed a significant and dose-dependent increase in ambulatory activities for the mutant mice treated with either drugs compared to WT control littermates (amphetamine, Supplementary Fig. 1A; methylphenidate, Supplementary Fig. 1B). Total activities over 90 min were shown for amphetamine (2.0 mg/kg) (Fig. 4A) and methylphenidate (2.5 mg/kg) (Fig. 4C). In response to amphetamine, *zdhhc15*-KO mice showed significantly increased activities compared to control during the first 60 min but comparable activities to controls during 60–90 min (Fig. 4A). In response to methylphenidate, *zdhhc15*-KO mice showed significantly increased activities compared to control during entire 90 min (Fig. 4C). We measured extracellular DA levels in the ventral striatum of *zdhhc15*-KO and control mice treated with 2 mg/kg of amphetamine by in vivo microdialysis. Results showed an increase in the extracellular levels of DA after amphetamine treatment in both groups but with a significantly higher level in the KO compared to WT mice (Fig. 4B). These data suggest that *zdhhc15* deficiency in mice confers an enhanced sensitivity to psychostimulants as reflected by increased activities and extracellular DA levels in ventral striatum, and the enhanced responses correlate with increased extracellular DA levels implicating altered DA release and/or synaptic clearance in the ventral striatum.

### No change in key proteins in DA signaling or metabolism in *zdhhc15*-KO mice

**Fig. 5:**
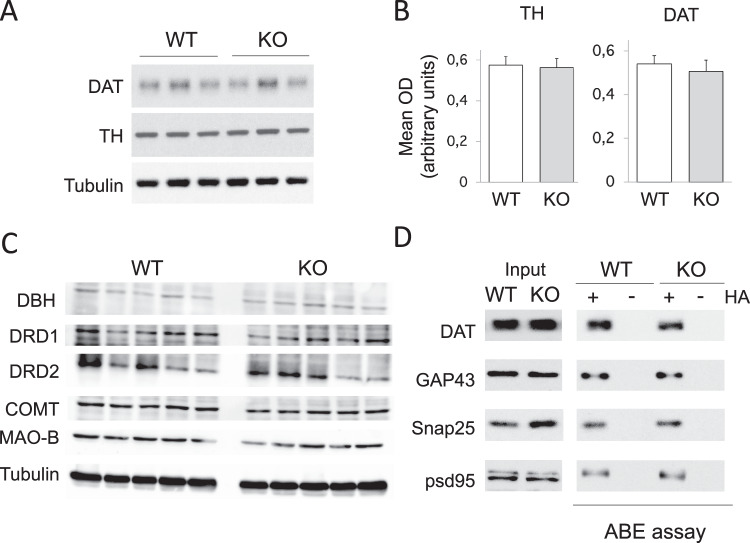
Analyses of zdhhc15 substrates and proteins involved in DA metabolism in striatum of *zdhhc15*-KO mice. **A**, **B** Western blot analyses of DAT and TH in striatal proteins extracts from *zdhhc15*-KO and WT mice revealed no significant differences between the two genotypes (*n* = 10 animals per group, student *t*-test). **C** Levels of other proteins involved in DA signaling and degradation were similar between WT and KO mice (*n* = 5 animals per group; student *t*-test, *p* > 0.05). **D** Acyl-biotin exchange (ABE) assay was performed for quantitative analysis of protein palmitoylation in striatum from WT and *zdhhc15*-KO mice. Representative blot signals of inputs (left) and ABE samples (right) in striatum of *zdhhc15*-KO and WT mice are shown. No differences were found in the total level of proteins or in the palmitoylation levels of known DHHC15 substrates (*n* = 5–6 animals per group; student *t*-test, *p* > 0.05).

Because DA alterations in *zdhhc15*-KO mice could be caused by changes in proteins involved in DA signaling and/or metabolism, we therefore quantified key protein levels in the striatum of WT and KO mice by Western blot analysis. We found no significant differences in dopamine transporter (DAT), tyrosine hydroxylase (TH), dopamine beta hydroxylase, D1 dopamine receptor (DRD1), D2 dopamine receptor (DRD2), catechol-O-methyltransferase (COMT), or monoamine oxidase B (MAO-B) levels in striatum of *zdhhc15*-KO mice and WT littermates (Fig. 5A–C; Supplementary Fig. 2A).

### No change in basal palmitoylation of known zdhhc15 substrates in *zdhhc15*-KO mice

We studied basal palmitoylation levels of known ZDHHC15 substrates (DAT, GAP43, psd95, and SNAP25) in striatal tissues from *zdhhc15*-KO mice and WT littermates using an ABE assay. No differences in basal palmitoylation levels of these substrates were found in striatum of *zdhhc15*-KO mice compared to WT (representative samples in Fig. 5D; levels of basal DAT palmitoylation, Supplementary Fig. 2B). Furthermore, comparable levels of basal palmitoylation were found for known ZDHHC15 substrates in parietal cortex tissues between *zdhhc15*-KO mice and WT littermates (Supplementary Fig. 3).

## Discussion

*Zdhhc15*-KO mice were found to have a significant increase in ambulatory activities in open field. Specifically, this hyperactivity phenotype was observed during the first 30 min or the habituation period in the test chamber and gradually reduced to baseline levels over 60–90 min, suggesting that the increase in locomotion is novelty-induced. Neurochemistry analyses show that while the dopamine content in striatal tissues was reduced, there was a significant increase in its extracellular DA levels corresponding to the period of increase in novelty-induced locomotion.

These behavioral and striatal neurochemical profiles of *zdhhc15*-KO mice are reminiscent of mice lacking dopamine transport (DAT). *Dat*-deficiency in mice impairs DA reuptake at the synapses, reduces extracellular DA content, rises extracellular DA levels, and significantly increases ambulatory activity in open field^56^. Previous studies have shown that DAT is palmitoylated and defects in palmitoylation reduce transporter stability and functions^35^. At least five PATs including ZDHHC15 were shown to increase palmitoylation of DAT using in vitro assays^34^. We speculate that *zdhhc15* deficiency could contribute to palmitoylation defects of DAT, which results in a transient and reversible reduction of DA reuptake at the synapses during the habituation period to a novel environment (open field). Reduced DAT function results in an increase extracellular DA levels, and reduction of striatal DA content. This is a highly regulated process that is consistent with the transient and reversible nature of palmitoylation. This hypothesis is further supported by enhanced locomotion of *zdhhc15* KO mice in response to acute administration of amphetamine and methylphenidate. Both drugs are psychostimulants that are known to regulate DA signaling in striatum via direct and/or indirect modulation of DAT functions^58^.

However, there is a striking contrast in behaviors and neurochemistry between *zdhhc15*-KO mice and homozygous or heterozygous *Dat1*-KO mice^59^. After an initial period of neophobia in open field, homozygous *Dat1*-KO mice display an anxiety-like response and a persistent increase in stereotyped locomotion that is unrelated to exploration or novelty seeking. These mutant mice maintain a ~5-folds elevation of extracellular DA levels from WT baseline and the levels exhibit no change at the time of stereotypic activation^59^. Heterozygous *Dat1*-KO mice display no significant difference in their total open-field activity. These mice show enhanced anxiety with increased time spent in the center of an open field and exhibit a moderately enhanced investigation of novel objects^59^. These mice maintain a ~2-folds elevation of extracellular DA levels from WT baseline^59^. These data suggest that novelty-induced hyperactivity is a highly regulated process that requires an inducible and reversible elevation of extracellular DA levels in response to a novel environment or stimulus rather than a persistent elevation of extracellular DA levels in striatum. Mechanisms regulating novelty-seeking behavior are largely unknown. Further studies of ZDHHC15- mechanisms responsible for novelty induced, transient and reversible changes of DAT function and/or other proteins involving in DA metabolism and signaling are warranted.

Excessive extracellular DA is subjected to degradation by COMT and/or MAO, which could contribute to the reduced total striatal DA content in *zdhhc15*- and *dat1*-KO mice^59^. Furthermore, an increase in extracellular DA at the synapses is known to inhibit DA synthesis by overstimulation of D2-autoreceptors and PKA-mediated phosphorylation of TH^60^. Because changes in extracellular DA levels can also be caused by altered DA metabolism, we conducted immunoblot to quantify key proteins involved in DA signaling and/or metabolism: tyrosine hydroxylase (TH), dopamine beta hydroxylase (DBH), DRD1, DRD2, COMT, MAO-B, and DAT. This study identified no significant changes of these proteins in striatum of *zdhhc15*-KO mice, suggesting mechanisms other than changes in steady-state levels of key DA regulatory proteins in striatum are potentially responsible.

It is intriguing that these studies did not detect a significant change in basal palmitoylation levels of DAT and other known ZDHHC15 substrates in striatal tissues between *zdhhc15*-KO and WT mice using a standard ABE assay. Palmitoylation is a dynamic, reversible, and regulated process while the standard ABE assay is designed to quantify the steady-state levels of an individual palmitoylated substrate. There are considerable levels of redundancy and complementation of established PATs for their substrates^30^. It has been shown that one PAT can palmitoylate several substrates and multiple PATs contribute to palmitoylation of a single substrate^30^. For example, ZDHHC15 was shown to palmitoylate PSD95, GAP43, CSP, Sortilin, Stathmin-2, and DAT^34,47^. PSD95 palmitoylation can be enhanced by ZDHHC-2, -3, -7, and -15 and DAT by ZDHHC-2, -3, -8, -15, and -17^30,46^. Furthermore, most PATs and their substrates were established by co-expression of constructs for individual PATs with the candidate substrate in heterologous cells^34,46^ and substrate specificity for these PATs has not been validated in vivo^22^ It is conceivable that standard ABE assay may not have sensitivity and specificity to capture a transient and reversible palmitoylation of a given substrate during the critical period of changing stimulation. Quantitative analysis of striatal palmitome in *zdhhc15*-KO mice and multiple KO mice for redundant PATs^47,61^, as well as developing novel in vivo assays to detect dynamic palmitoylation would help to characterize underlying mechanisms of ZDHHC15-palmitoylation in modulating dopaminergic phenotype in *zdhhc15*-KO mice.

Novelty seeking refers to behaviors including exploratory excitability, impulsive decision-making, and quick loss of temper^7^. It is a core dimension of temperament defined as an individual profile of biological response to the environment^7^. Novelty-seeking behavior is predictive of high inattention and hyperactivity–impulsivity symptom scores, two core behavioral domains in both children and adults with ADHD disorders^9,12^. A strong genetic correlation was found between hyperactivity–impulsivity and novelty seeking in a pediatric twin study^62^. A recent study of 866 pairs of adult ADHD twins of 19–20 years showed that novelty-seeking is genetically associated with both inattention and hyperactivity–impulsivity, two core ADHD symptom dimensions^15^.

Previous studies have shown that dopamine neurons increase their firing rate in response to stimulus of novelty^63,64^ and novelty signals originating in the hippocampus modulate the activity of dopamine neurons in substantia nigra/ventral tegmental area (SN/VTA)^18^. Data from high-resolution functional magnetic resonance imaging (fMRI) support that neural responses to novelty seeking is within the SN/VTA complex in humans^19^. Dopamine D4 receptor gene (DRD4) has been associated with novelty seeking in humans^65,66^ though its definitive role remains to be established^67^. Previous studies of a high novelty responsive rat (HR) model identified a distinct profile of DA metabolites and a higher basal and extracellular dopamine levels in the nucleus accumbens^16,17^ supporting that disturbances in DA metabolism and/or signaling play an important role in novelty seeking in rodents. Further characterization of ZDHHC15-palmitoylation of DAT and other DA-signaling proteins in striatum should shed lights on regulatory mechanisms of novelty seeking and ADHD.

An X-autosome translocation disrupting *zdhhc15*-expression was reported in a female patient with intellectual disability^48^. Intriguingly, another female patient with a different X-autosome translocation interrupting *ZDHHC15* expression was reported to only have primary amenorrhea without cognitive impairment^50^. A zebrafish model for mutant *zdhhc15b* showed impairment in learning and memory behaviors in a T-maze test as well as reduced number of dopaminergic neurons and levels of TH and DAT proteins^44^. *Zdhhc15* knockdown in cultured rat hippocampus neurons by shRNA was found to inhibit dendritic outgrowth and formation of mature spines^45^. *Zdhhc15*-KO mice show normal number and projections of dopaminergic neurons in the nigrostriatal and mesolimbic pathways. *Zdhhc15*-KO mice did not manifest severe deficits in spatial learning, working memory, or fear memory in standard rodent behavioral testing. It is conceivable that ZDHHC15 could have diverse functions depending on the species, and that acute knockdown of *zdhhc15* transcript in neurons may not fully recapitulate potential compensatory effects associated with lack of ZDHHC15-palmitoylation during early brain development. Further studies are needed to establish the roles of genetic defects in *zdhhc15* in cognitive disorders in humans^49^.

## Supplementary information

Supplemental Materials
