# Supplementary material for: Increased novelty-induced locomotion, sensitivity to amphetamine, and extracellular dopamine in striatum of Zdhhc15-deficient mice
Source: Transl Psychiatry. 2021 Jan 18;11:65. doi: 10.1038/s41398-020-01194-6 (PMC7813841; doi:10.1038/s41398-020-01194-6)
Supplement: Supplementary file 1 — Supplemental Materials [file 41398_2020_1194_MOESM1_ESM.pdf]

Supplemental Materials

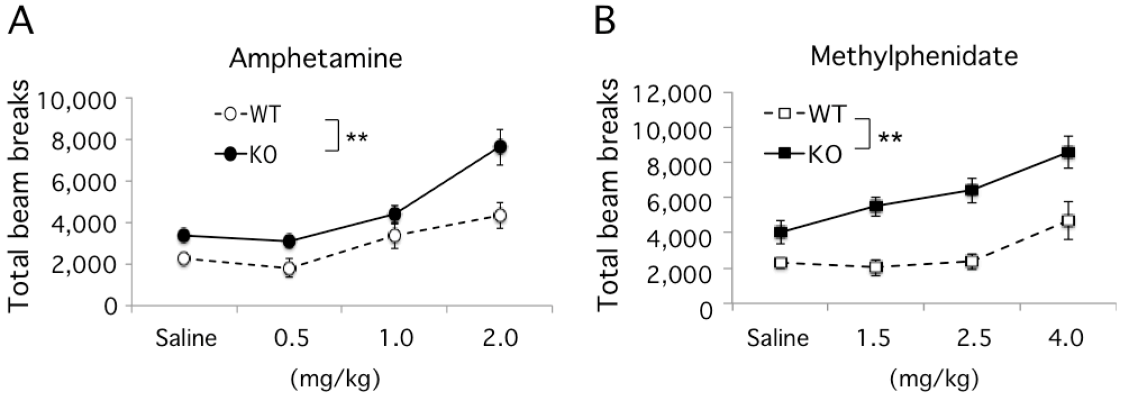

Supplementary Figure 1. Dosage-responsive Ambulatory Activities of *Zdhhc*15-KO Mice to Amphetamine and Methylphenidate. **A.** Total ambulatory activities of *zdhhc*15-KO mice in open field (30 minutes) after i.p. injection of normal saline and amphetamine at the indicated doses (0.5, 1.0, 1.5 mg/kg). **B**. Total ambulatory activities of *zdhhc*15-KO mice in open field (30 minutes) after i.p. injection of normal saline and methylphenidate at the indicated doses (1.5, 2.5, 4.0 mg/kg). Noted dose-dependent increase in activities in *zdhhc*15-KO and WT control mice in response to amphetamine and methylphenidate, respectively. Significant differences were detected between WT and *zdhhc*15-KO mice. Total number of animals for *zdhhc*15-KO mice and WT controls: n=9-10 per study group. Mean and SEM of total beam breaks were shown. **, Factorial ANOVA analysis, $p<0.01$.

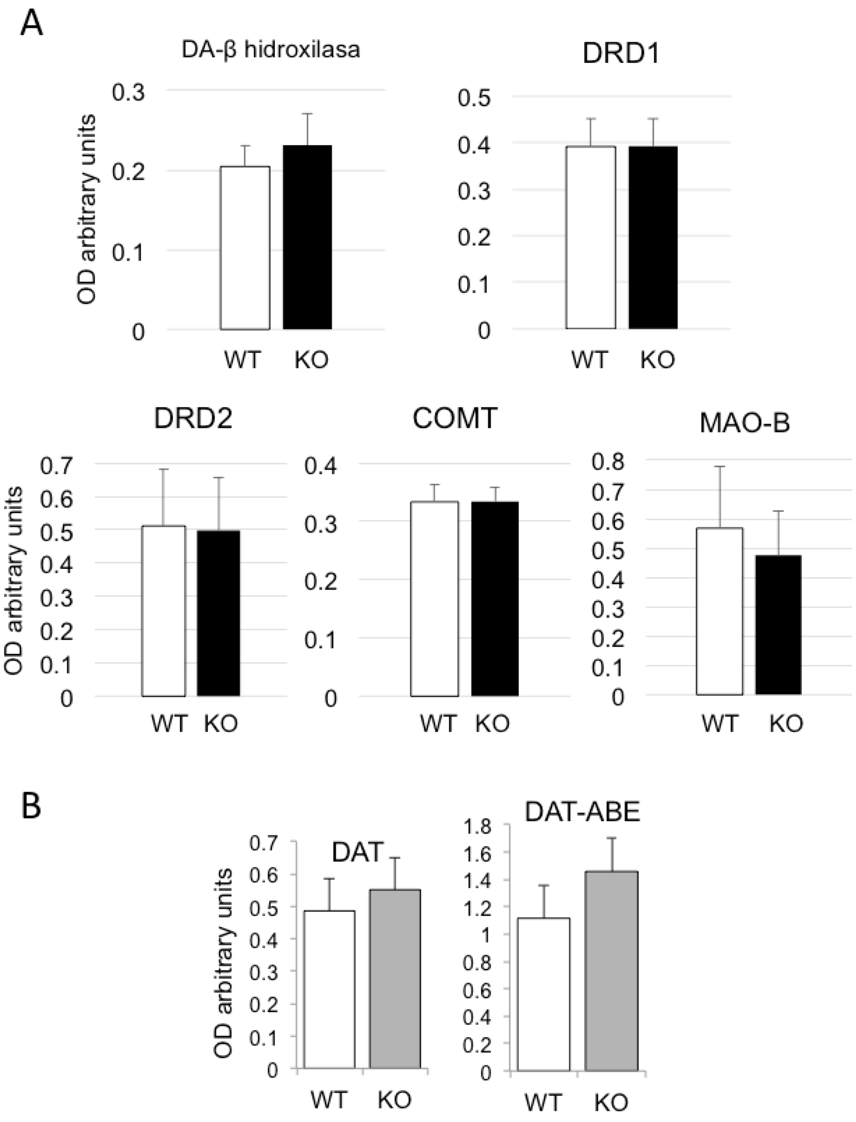

Supplementary Figure 2. Quantitation of the total and palmitoylation levels of proteins involved in DA metabolism in the striatum of *zdhhc15*-KO mice. **A.** No differences were observed in the protein levels of enzymes involved in signaling and degradation of DA quantified by western blot analyses (n=5 animals per group, student t test; $p<0.05$). **B.** Quantification of input signal and palmitoylation levels of DAT in the striata of KO and WT mice showing no differences between the two groups (n=5-6 animals per group, student t-test, $p>0.05$).

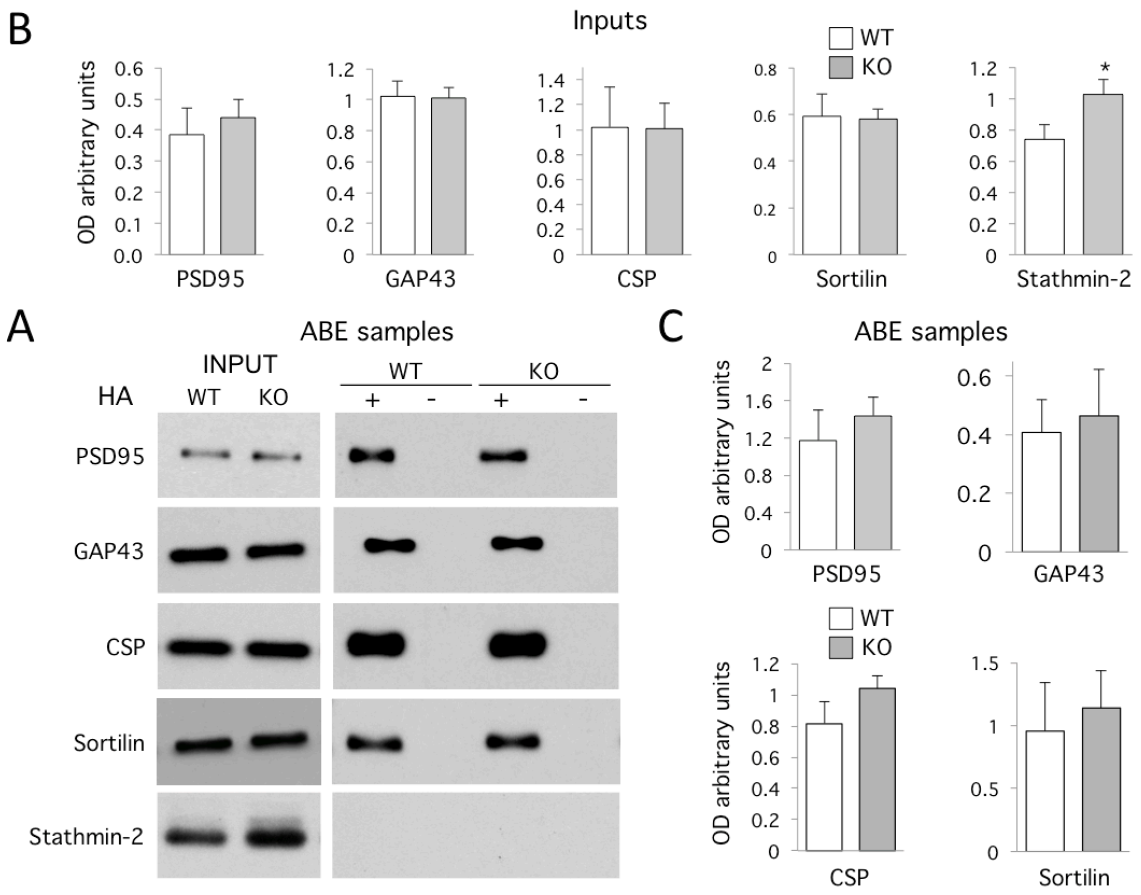

Supplementary Figure 3. Analysis of palmitoylation levels of known substrates of zdhhc15 in parietal cortex of KO and WT mice. **A.** Representative blot signals of inputs (left) and ABE samples (right) for PSD95, GAP43, CSP, Sortilin and Stathmin-2 in parietal cortex of *zdhhc15*-KO and WT mice are shown. Palmitoylation levels of Stathmin-2 were under our limit of detection. HA, hydroxylamine. Blot signals of inputs **B.** and ABE samples **C.** in brain cortex were quantified using ImageJ and normalized to input. No differences were found in the palmitoylation levels of ZDHHC15 substrates in WT versus KO mice or in the total levels of the substrates except for a mild increase in the total amount of Stathmin-2 (n=5-6, student t test, p=0.044 for Stathmin-2 input).

Supplementary Table 1. Behavioral Characteristics of Male Z*dhhc15*-KO and WT Littermates

	WT	*Zdhhc15*-KO
Elevated plus maze		
% Time in closed arms	50 ± 4	44 ± 3
% Time open arms	34 ± 3	39 ± 3
Fear conditioning		
Total amount of freezing time (s)#	91.2 ± 11.7	85.7 ± 16.5
Pre-pulse inhibition		
Vmax / weight (mV/g)		
Pre-pulse intensity (0 dB)	11.2 ± 2.0	10.9 ± 2.2
Pre-pulse intensity (74 dB)	9.5 ± 2.1	8.5 ± 2.1
Pre-pulse intensity (78 dB)	7.1 ± 2.0	6.6 ± 2.0
Pre-pulse intensity (82 dB)	5.3 ± 1.4	4.7 ± 1.4
Pre-pulse intensity (86 dB)	4.0 ± 1.2	3.1 ± 0.9
Pre-pulse intensity (90 dB)	3.0 ± 1.0	2.7 ± 0.8
Morris Water Maze		
Time in right quadrant (s)	41.0 ± 2.5	41.8 ± 2.7
Y-Maze (spontaneous alternations)		
Total entries	14.9 ± 0	12.4 ± 1.2
% alternation / entries	55.4 ± 3.8	61.3 ± 5.7
Y-Maze (novel arm entries)		
% Time in novel arms (first 2 min)	33.4 ± 4.9	39.9 ± 4.8
% Time in novel arms (total 5 min)	34.2 ± 3.8	39.2 ± 3.7
Rotarod		
Latency to fall (s)	46.5 ± 2.9	39.5 ± 3.2
Sociability test		
Time interacting with empty cage (s)	69.6 ± 7.1	69.6 ± 11.1
Time interacting with stranger mouse (s)	140.1 ± 16.3	145.9 ± 10.1
Preference for social novelty		
Time interacting with familiar mouse (s)	79.8 ± 8.0	78.6 ± 6.6
Time interacting with stranger mouse (s)	98.1 ± 12.2	94.9 ± 10.1
Novel Object Recognition		
Time exploring familiar object (s)	9.7 ± 1.6	6.9 ± 1.2
Time exploring novel object (s)¶	12.6 ± 2.0	10.6 ± 1.8

Data are expressed as means ± SEM. Genotype-based comparisons were performed using Student's t-test for independent samples or two-way ANOVA (*p<0.05, **p<0.01, n=14-18 animals per group). # Data from trial 2 obtained 24h after trial 1; ¶ Data obtained 1 h after exploring 2 identical objects.

Supplementary Table 2. Monoamine Concentrations in Selected Brain Regions of Z*dhhc15*-KO and WT Mice

Brain Regions	Monoamine Family	Monoamine	WT	*zdhhc15*-KO
			Concentration (pg/mg tissue)	
Ventral Mesencephalon	Norepinephrine	NE	402.27 ± 23.91	419.75 ± 20.40
	Dopamine and metabolites	DA	224.34 ± 16.82	193.18 ± 16.08
		DOPAC	130.82 ± 6.21	132.52 ± 8.60
		HVA	236.69 ± 6.15	238.92 ± 16.94
	Serotonin and metabolites	5-HT	1769.9 ± 69.60	1727.36 ± 87.80
		5-HIAA	1499.37 ± 81.94	1558.66 ± 129.65
Prefrontal Cortex	Norepinephrine	NE	35.68 ± 3.07	38.92 ± 1.88
	Dopamine and metabolites	DA	63.75 ± 4.04	54.10 ± 4.57
		DOPAC	67.02 ± 11.40	53.14 ± 6.78
		HVA	127.93 ± 14.48	125.02 ± 14.63
	Serotonin and metabolites	5-HT	630.14 ± 37.40	648.51 ± 27.29
		5-HIAA	257.66 ± 18.42	287.20 ± 19.24
Olfactory Bulb	Norepinephrine	NE	172.9 ± 10.31	165.58 ± 15.29
	Dopamine and metabolites	DA	232.06 ± 15.44	212.84 ± 20.80
		DOPAC	36.69 ± 6.62	34.2 ± 6.97
		HVA	203.82 ±14.98	217.88 ± 20.22
	Serotonin and metabolites	5-HT	553.49 ± 14.23	596.94 ± 21.13
		5-HIAA	251.99 ± 11.11	291.54 ± 14.01*

NE, norepinephrine; DA, dopamine; DOPAC, 3,4-Dihydroxyphenylacetic acid; HVA,Homovanillic acid; 5-HT, 5-hydroxytryptamine or serotonin; 5-HIAA, 5-hydroxyindoleacetic acid. Data are expressed as means ± SEM. Genotype-based comparisons were performed using Student's t-test for independent samples. (*$p<0.05$, n=9-10 samples per group).
